# A high diversity naïve variable new antigen receptor, vNAR, phage library for rapid nanobody discovery across diverse antigens

**DOI:** 10.1016/j.jbc.2025.111083

**Published:** 2025-12-22

**Authors:** Vivek Kumar, Kuldeep Jangid, B. Santhosh, Riya Dixit, Urvashi Yadav, Surekha Verma, Archana Rout, S. Surya, Manjima Das, Rohit Gupta, Anjali Saroj, Rishav Madhukalya, Megha Gupta, Muskan Kalra, Hiba Iqbal, Dilip Kumar, Subrata Sinha, Shailly Tomar, Pravindra Kumar, Rajesh Kumar

**Affiliations:** 1 Department of Biosciences and Bioengineering, Indian Institute of Technology, Roorkee, Uttarakhand, India; 2 Mariculture Division, Vizhinjam Regional Centre of Central Marine Fisheries Research Institute, Thiruvananthapuram, Kerala, India; 3 Trivedi School of Biosciences, Ashoka University, Sonipat, Haryana, India; 4 Department of Biochemistry, All India Institute of Medical Sciences, New Delhi, India

**Keywords:** high-throughput screening platform, naïve library, universal single pot vNAR library, variable new antigen receptor

## Abstract

Conventional antibodies are among the most frequently used and effective biological tools explored for therapeutic and diagnostic applications. However, they face significant limitations when it comes to challenges that demand specialized attributes such as rapid tissue penetration, the ability to bind to concealed epitopes, and stability in nonphysiological environments. In recent years, shark-derived immunoglobulin variable new antigen receptor (vNAR) has emerged as a promising alternative to overcome these limitations. In this study, we constructed a naïve vNAR phage display library from a white-spotted bamboo shark (*Chiloscyllium plagiosum*), with a library diversity size of ∼3 × 10^11^ clones. Next generation sequencing analysis revealed the high diversity of the library, allowing it to encompass a broad range of classical functional vNAR types. To confirm the usability of the library for the successful isolation of positive clones, we screened the library against wide range of antigens (n = 9;) from different origin that includes viral, cancer, autoimmune, toxins, parasite, algae, and plant antigens. We achieved a hit rate of ∼100%, of potent binders with micro to nanomolar range affinity. The total number of unique binder’s clones varied from 30%-100%, depending on the antigens and screening strategy. Furthermore, we provide an in-depth structural analysis by using X-ray crystallography of class IV vNARs from bamboo sharks, which remain underexplored. Our study represents a significant step forward in the field of single-domain antibody research and development.

The high affinity and specificity of therapeutic antibodies have driven the development of newer technology and significant advancements in the field of immunology and biotechnology. Despite their extensive characterization and prevalent scientific interest, the usage of standard antibodies like immunoglobulin G (IgG) remains limited in certain biomedical areas due to persistent challenges associated with it, such as large size (150 kDa), complex structural features, high production costs, and difficulty in effectively accessing certain antigens or cryptic epitopes (1). To address these challenges and to advance therapeutic potential, consistent efforts have been made in engineering the next-generation antibodies, nonimmunoglobulin-based protein scaffolds, and antibody fragments as optimal alternatives. Single-domain antibodies (also known as “nanobodies” or VHH) are one of the most promising candidates (2), (https://pmc.ncbi.nlm.nih.gov/articles/PMC10057852/). Nanobodies, which consist of a single monomeric variable domain, were initially discovered in camelids. Subsequently, similar structures known as vNARs were reported in cartilaginous fish, specifically sharks. Shark-derived vNARs are a vital tool in the therapeutic arsenal due to their unique properties, such as small size (∼12–50 kDa), which facilitates greater tissue penetration and enables binding to inaccessible protein clefts and functional sites. At the same time, vNARs retain their solubility and stability in harsh environments involving low pH, high urea concentrations, and elevated temperatures, typically exceeding the thermal stability threshold of scFvs and mAbs (3). These attributes confer a significant advantage to vNARs over traditional antibodies. Another key feature of vNARs is their extended complementarity-determining region 3 (CDR3), which consists of 9 to 34 amino acids. The CDR3 loop exhibits exceptional sequence diversity and forms a variety of structural topologies, including distinct disulfide-bridge patterns (4, 5, 6). This feature grants them the unique ability to form finger-like extensions, enabling access to cryptic and/or cleft-like epitopes on target proteins that are typically inaccessible to conventional antibodies. Structural studies have revealed four distinct types of vNARs (type I-IV) based on the number and positions of noncanonical cysteine residues and their corresponding disulfide bond arrangements. The vNAR domain consists of two β-sheets held together by canonical cysteine residues, along with additional hypervariable regions (HV2 and HV4) that contribute to its unique architecture and binding properties (3, 7). Due to distinct structural and functional characteristics, vNARs have emerged as promising candidates for diverse biomedical applications. However, to fully harness the potential of shark vNARs, it is essential to develop diverse vNAR libraries that can be screened against a wide range of target molecules with varying binding properties. In a related study, Mingquain *et al.* constructed a large naïve vNAR library from six nurse sharks, achieving a total library size of ∼ 1.2 × 10^10^ unique clones (7). In our current study, we further advance this work by creating a naïve vNAR library from the bamboo shark (*Chiloscyllium plagiosum*) having 10^11^ functional clones. The bamboo shark presents specific advantages as an animal model for vNAR production due to its small size, sedentary nature, and suitability for large-scale husbandry, making it an ideal candidate for creating diverse, high-quality vNAR libraries. We further screened this library against diverse panel of antigens (n = 9) including antigens of viral (n = 2), plant (n = 1), cancer (n = 2), autoimmune (n = 1), parasite (n = 1), toxin (n = 1), algal (n = 1) origin and identified high affinity binders from micromolar to nanomolar affinity. We also developed a high throughput screening system where we can develop vNAR nanobodies in two to 3 weeks’ time against any given antigens. Additionally, we expressed, purified, and performed detailed structural analysis of type IV vNARs from our library. Our structural analysis enables us to map essential residues involved in antigen interaction, allowing more empirical design and engineering of vNARs with enhanced specificity and affinity. Overall, our study is a crucial step towards optimizing vNARs and exploring their full potential as next-generation antibody alternatives in challenging biomedical contexts.

## Results

### Construction of a highly diverse naïve vNAR phage library

**Figure 1:**
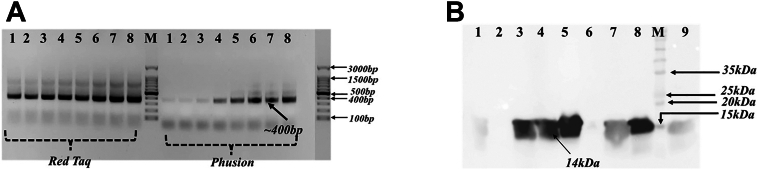
**PCR amplification and functional validation of vNAR.***A*, agarose gel electrophoresis (1.5%) depicting PCR amplification of vNAR sequences using Taq DNA polymerase (*left* panel) and with Phusion polymerase (*Right* panel). Lane M: 100 bp DNA ladder; lanes one1 to 8: gradient annealing temperatures ranging from 55 °C to 62 °C used for PCR optimization. A cropped image of 100 bp ladder has been labeled with arrow mark showing molecular size of different bands in DNA ladder. *B*, Western blot analysis of periplasmic extracts from BL21 (DE3) cells expressing vNAR-phagemid constructs. Lane 1 to 9: randomly selected vNAR-expressing clones; lane M: protein molecular weight marker. vNAR, variable new antigen receptor.

To generate a large naïve vNAR library with high diversity, spleen samples from two bamboo sharks were processed, and spleen cells were isolated. The isolated spleen cells were resuspended in Trizol solution, and a total of 50 μg of RNA was extracted from the spleen cells to generate complementary DNA (cDNA). The quality of the RNA sample was assessed by measuring an absorbance ratio of A260/A280 (∼2.0) to ensure high purity. To minimize cDNA production bias in our experiment, we used a freshly prepared RNA sample for cDNA synthesis and avoided freeze-thaw cycles after RNA extraction. Additionally, we used an optimized primer mix that contains a combination of random hexamers (for ensuring a uniform representation of the entire transcript coverage) and oligo(dT) primers (for full-length transcripts) to cover across the transcript length. The vNAR genes were PCR-amplified using specifically designed forward and reverse primers that carry the restriction site for *NcoI* and *NotI*, respectively. Gel electrophoresis of the amplified vNAR sequences showed a distinct band of ∼400 bp (Fig. 1*A*). PCR reactions were performed using both Phusion polymerase and Taq DNA polymerase. Gradient PCR amplification showed that Taq DNA polymerase was efficient at all the tested annealing temperatures (55 °C to 62 °C); however, the amplification efficiency of Phusion polymerase dropped at higher annealing temperatures (Fig. 1*A*). Additionally, Taq polymerase maximized the diversity of the amplified vNAR genes as it incorporated random mutations during amplification. Based on the results of gradient PCR and due to the inherent ability of Taq DNA polymerase to add new mutations, Taq polymerase was preferred over Phusion polymerase to carry out the bulk PCR amplification of the vNAR genes (8). Although amplification bias increases with the number of cycles and preferably under 25 cycles helps in maintaining a more uniform representation of the original cDNA pool. However, reducing the number of PCR cycles to 25 resulted in faint bands and an overall lower yield of amplified vNAR genes. In our experiments, we found that a minimum of 32 PCR cycles resulted in an optimal amplification of vNAR genes.

The PCR-amplified vNAR fragments and the pSEX81 phagemid vector were digested using *NcoI* and *NotI*, and subsequently, two separate ligation reactions were carried out with vector-to-insert ratios of 1:1 and 1:2, respectively, in order to determine the optimum insert volume needed for efficient ligation. Fragments ligated at a vector-to-insert ratio of 1:1 exhibited higher transformation efficiency, resulting in a higher number of transformants upon plating than those ligated at a vector-to-insert ratio of 1:2 (Fig. S1). The optimum time required for successful ligation was ascertained by performing the ligation reaction with a vector-to-insert ratio of 1:1 for two time intervals: 1 h at room temperature and overnight at 16 °C. Ligated fragments from the overnight ligation reaction yielded a higher number of transformants than the 1 h ligation reaction (Fig. S2). Thus, the vNAR library was constructed using the optimized ligation parameters, which included a 1:1 vector-to-insert ratio and an overnight incubation period. Bulk ligation reactions were performed with 3 μg each of digested vector and insert. The ligated fragments were transformed into electrocompetent TG1 cells. TG1 cells were incubated for a maximum of 40 to 50 min after the electric shock to avoid the repetition of similar types of vNAR clones. This process was repeated 15 times, producing 15 sublibrary stocks, each with a size of ∼2 × 10^10^. Finally, all 15 sub-library stocks were pooled together to form a central library with a size of ∼3 × 10^11^ (Fig. S3). The background was determined in parallel by transforming the digested phagemid vector into TG1 cells.

A common challenge in the panning process is caused by the presence of phage with incomplete or defective vNAR sequences. To calculate the percentage of phage with full-length vNAR sequences, 100 clones were randomly selected from each sublibrary stock (∼2 × 10^10^) and analyzed *via* colony PCR (Fig. S4) and restriction digestion using *Nco1* and *Not1*. Notably, >97% (97/100) of the clones in the phage library were found to contain complete vNAR sequences (Fig. S5).

### Functional validation of the constructed vNAR central library

**Figure 2:**
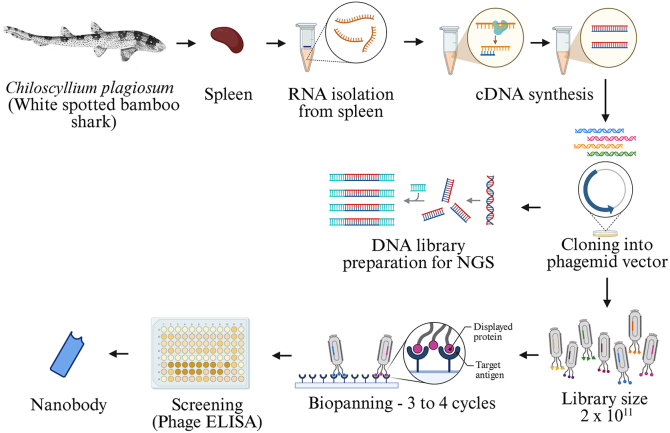
**A schematic representation of the different steps involved in naïve vNAR phage library construction.** The spleen cells were isolated from white-spotted bamboo sharks *(Chiloscyllium plagiosum).* Total RNA was isolated and complementary DNA was synthesized. vNAR genes were PCR-amplified and cloned into a phagemid vector. The vNAR phage library of 2 × 10^11^ unique clones was constructed. vNAR, variable new antigen receptor.

Functionality and diversity of the vNAR clones are the major factors that determine the effectiveness of a naïve library. To test the functionality of the clones, plasmids from 20 randomly selected clones were isolated from each sublibrary, resulting in the selection of ∼300 clones. The isolated plasmids were retransformed into the *Escherichia coli* BL21 (DE3) strain, and the production of soluble vNAR antibodies was induced with IPTG. Subsequent to the induction period, cells were chemically lysed and analyzed on SDS-PAGE. Upon SDS-PAGE analysis of the soluble cell fractions, distinct protein bands corresponding to ∼14 kDa were observed and further confirmed through Western blot (Fig. 1*B*). The expression profile showed that >85% of clones were positive for vNAR expression (255/300), thereby confirming the functionality of the vNAR central library (Fig. S6). Schematic representations of the steps involved in our library construction are shown in Figure 2.

### Sanger sequencing and NGS analysis to validate library diversity

**Figure 3:**
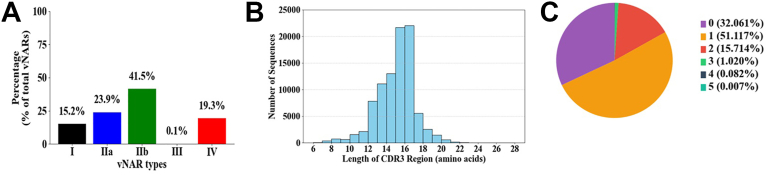
**NGS analysis of naïve vNAR library.***A*, distribution of total 238,415 unique vNAR sequences within the library. Type I vNARs (36,256 sequences, ∼15%) contain an even number of Cys residues within the CDR3 region. Type IIa vNARs (57,112 sequences, ∼24%) contain Cys residues in both CDR1 and CDR3. Type IIb vNARs (98,849 sequences, ∼41%) have Cys in either CDR1 or CDR3, but not in both. Type III vNARs (8 sequences, <0.1%) closely resemble type II vNARs except that they include a conserved Trp residue in the CDR1 region. 46,190 sequences (∼19%) from total sequences were analyzed as type IV vNARs. *B*, distribution of CDR3 lengths in the vNAR clones, ranging from 6 to 28 amino acids, with ∼50% of the clones having lengths of 15 to 16 amino acids. *C*, proportion of CDR3 sequences with different numbers of Cys residues among ∼91,600: 46,823 CDR3 sequence (∼51%) contain one Cys, 14,395 CDR3 sequence (∼15%) contain two Cys, 934 sequence (∼1%) contain 3 Cys, and 29,367 CDR3 sequence (∼32%) contain no Cys residues. CDR, complementarity-determining region; vNAR, variable new antigen receptor.

Sanger sequencing was carried out on 30 randomly selected clones to validate the diversity of the library. The sequencing data confirms that >90% (45/50) of the clones were unique and primarily harbored vNAR genes that belonged to type IIb and type IV vNAR variants. To further validate these findings across a larger subset of the library, randomly selected clones from our vNAR library were sequenced using high-throughput paired-end sequencing methods (Fig. S7). The raw sequencing data revealed ∼0.2 million (238,415) unique vNAR protein sequences, with 57,112 (∼23.9%) sequences containing conserved Cys residues that are involved in interloop disulfide linkages between CDR1 and CDR3-a hallmark of typical type II (type IIa) vNAR. In contrast, 98,849 (∼41.5%) sequences were classified as atypical type II (type IIb) vNAR, lacking a cysteine in either CDR1 or CDR3 and thus unable to form the interloop disulfide linkage. The absence of this bond results in more flexible loop structures in type IIb vNAR than the type IIa vNAR, resulting in a topology similar to that of type IV vNAR. Thus, type IIb vNARs are also referred to as type IV domains according to Streltsov *et al.* and Liu *et al.* (9, 10). Around 36,256 (∼15.2%) sequences belong to type I vNAR as they showed an even number of Cys residues in the CDR3 region that enable the formation of intraloop or interloop disulfide linkages. Eight (<0.1%) sequences belong to type III vNAR, which contains Cys and Trp at the 29th and 30th positions, respectively, in the CDR1 region. The remaining 46,190 (∼19.3%) sequences were categorized as type IV vNAR (Fig. 3*A*). Sequence alignment further revealed that most of the type II vNAR sequences had a conserved Leu at the 31st position within the CDR1 region. Maximum variability was identified in the CDR3 region in terms of amino acid composition and length (ranging from 6 to 28 residues) (Fig. 3*B*). From the sequence comparison, we found that most of the conserved residues are present in the framework regions, and maximum variability lies in CDR3. Ninety one thousand six hundred (∼38%) vNAR sequences have unique CDR3 regions, and the remaining sequences have variability in other regions, such as FR and CDR1 regions. The number of cysteine residues in the CDR3 varied from 0 to 5 (Fig. 3*C*).

### Panning of naïve vNAR library against diverse set of antigens

**Figure 4:**
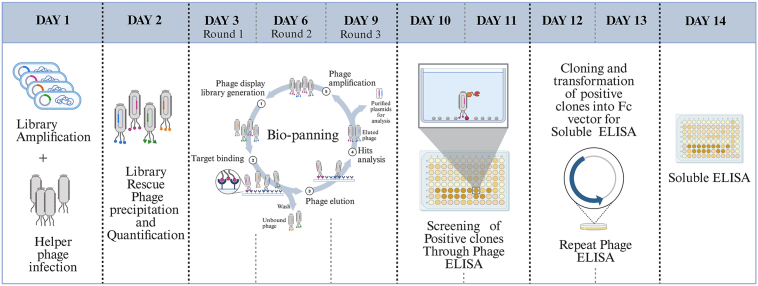
**Strategy for the isolation of antigen-specific vNAR clones.** The optimized biopanning strategy is established as a high-throughput screening platform spanning 14 days, starting from the availability of the purified antigen, followed by the preparation of the central vNAR library and helper phage stocks (days 1–2). Three rounds of panning against the desired antigen were conducted (day 3–9). Random clones were selected after BP2 and BP3 to screen positive binders (day 10–11). Typically, three rounds of biopanning yield a good number of unique sequence binders. Positive clones identified after BP2 and BP3 undergo three sets of independent phage ELISA assays to select consistent binders (days 12 and 13). Finally, selected binders are validated in soluble ELISA assays (day 14). vNAR, variable new antigen receptor; BP, biopanning.

**Figure 5:**
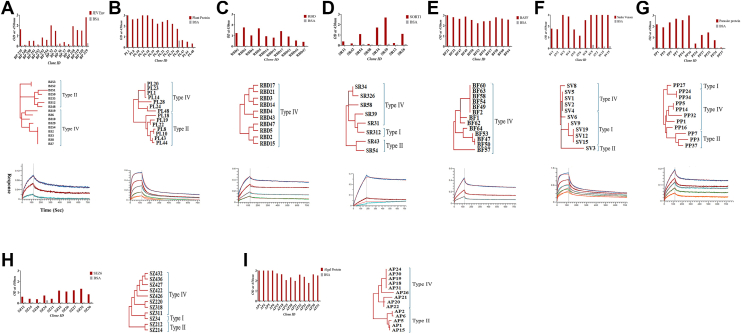
**Phage ELISA-binding specificity and phylogenetic diversity of positive binders.** Naïve vNAR library was biopanned against different antigens; (*A*) envelope antigen of JEV, (*B*) plant protein (5-enolpyruvylshikimate-3-phosphate, EPSP synthase from cotton plant), (*C*) RBD of SARS-CoV2, (*D*) breast cancer antigen (SORT1), (*E*) autoimmune antigen, BAFF (B-cell activating factor), (*F*) toxin from snake venom, (*G*) parasite protein (deoxyhypusine hydroxylase (DOHH) from *Leishmania donovani*), (*H*) lung cancer–specific antigen (seizure-related homolog protein 6, SEZ6), (*I*) algae protein crude extract. Panel 1 represents phage ELISA data. Clones showing ≥4-fold higher binding as compared to negative controls (milk and BSA) were considered positive. Phage ELISA assays were repeated ≥3 times to confirm consistent binding. Panel 2 represents the diversity of vNAR types among antigen-specific clones encompassed types I, II, and IV. Phylogenetic trees were constructed using MEGA 11. Panel 3 represents BLI data of nanobody clones with their respective antigens. For BLI experiments, randomly one clone was selected from each respective antigen except for SEZ6 and algae crude extract. For SEZ6, recombinant antigen was a limitation and for algae, we do not have the purified antigen to perform BLI. The x-axis represents time in seconds and y-axis represents response unit of association and dissociation. Different color in BLI data represents concentration of analyte. BLI, biolayer interferometry; vNAR, variable new antigen receptor.

The utility of a library depends on its ability to isolate antigen-specific vNARs against a diverse range of targets. We optimized the protocols for all the steps involved in biopanning. The overall turnaround time from antigen in hand to isolate antigen-specific vNAR is 2 to 3 weeks (Fig. 4). Two to four rounds of biopanning (BP) were performed. After each round of panning, approx. 30 to 50 clones were randomly selected and assessed for their binding with their respective antigens *via* ELISA. Clones showing a minimum of four-fold higher ELISA-binding signal as compared to negative antigens (bovine serum albumin [BSA], milk, and unrelated antigen) were considered phage ELISA positive. We observed that the number of positive clones isolated after each round of panning varied from antigen to antigen. However, on average, ∼1% of clones were antigen-positive (3/30) after the second round of BP, with the positivity rate increasing to ∼20% (6/30) in round 3 and ∼40% in round 4 (12/30). Similarly, the OD values of positive clones obtained after phage ELISA progressively increased as we moved from the second to the fourth round of BP. A mixed population of clones was observed in the third round of BP, displaying both high OD (>1.0) and medium OD (0.5–1.0) values. By the fourth round of BP, most of the phage ELISA-positive clones (∼70%) exhibited >1 OD values, indicating a significant enrichment of high-affinity binders (Fig. 5, *A*–*C*).

The binding specificity of the positive antigen-binding clones was further verified *via* soluble ELISA, where phage ELISA-positive clones were allowed to infect HB2151 *E. coli* cells. Unlike TG1, HB2151 cells are unable to suppress the amber stop codon located upstream of the gene III sequence in the phagemid vector, thus resulting in the expression of a nonfused nanobody without the pIII protein of phage. Notably, the majority of the phage ELISA-positive clones produced highly specific functional vNAR antibodies, with minimal or no cross-reactivity to other unrelated antigens (Fig. S8).

One of the major limitations for naïve libraries is considered as their selection of low-affinity binders (1). To validate this in our library, we determined the affinity of vNAR nanobodies isolated from our library against different target antigens using biolayer interferometry (BLI). Most of the tested vNAR nanobodies showed binding with micromolar to nanomolar affinity (Table 1). Interestingly, one of the nanobody clone targeted against breast cancer antigen (SORT1) showed a picomolar affinity binding. This data further verify that this library has a huge potential to be used as a universal source for the isolation of high affinity binders against diverse set of antigens (Fig. 5, *A*–*I*).

**Table 1:** Characteristics of antigen-binding clones and biopanning process against different antigens

S.No.	Antigen	Clones sent for sequencing	Unique clones	Percentage of unique clones	Affinity (Kd)
1	RBD	10	3	30%	2.87 × 10^-9^
2	JEV Env	16	12	75%	5.33 × 10^-8^
3	Breast Cancer (SORT1)	8	8	100%	3.8 × 10^-12^
4	Autoimmune antigen (BAFF)	13	7	53%	1.96 × 10^-8^
5	Parasite protein	11	9	81%	3.35 × 10^-8^
6	Plant protein	14	12	85%	1.14 × 10^-8^
7	Toxin (snake venom)	11	8	73%	3.81 × 10^-9^
8	Algal Protein	14	7	50%	Nd
9	Lung cancer (SEZ6)	10	7	70%	Nd

### Sequence variability in antigen-positive clones

To assess the sequence diversity of phage ELISA-positive binders, clones from each round of biopanning were selected and subjected to sequencing. Sequencing data showed that >90% of phage ELISA-positive clones selected after the second round of BP exhibited unique sequences. Similarly, 30 to 100% of the positive clones isolated from the third round of BP also showed uniqueness (Table 1). However, the proportion of unique clones decreased in the fourth round of BP, where ∼10 to 30% of the positive clones showed sequence diversity depending on the target antigen. Our results indicate that the constructed library can isolate a good number of unique binders against the various tested antigens, with the proportion of unique binding clones decreasing progressively from BP round 2 to round 4. Additionally, considerable variability was detected in the length of the CDR3 regions, ranging from 6 to 19 amino acid residues, which mirrors the CDR3 length distribution observed in the unselected naïve library. To better understand the diversity of our antigen-specific vNARs, we constructed phylogenetic trees using MEGA 11. The JEV Env-specific vNARs were primarily classified as type II and IV vNARs (Fig. 5). The vNAR against lung cancer, parasite protein, and venom toxin showed a wide range of distribution, including types I, II, and IV; RBD-specific vNARs were predominantly of type IV, vNARs against plant and algal antigen were of II and type IV (Fig. 5).

### The crystal structure of type IV vNAR nanobodies

**Figure 6:**
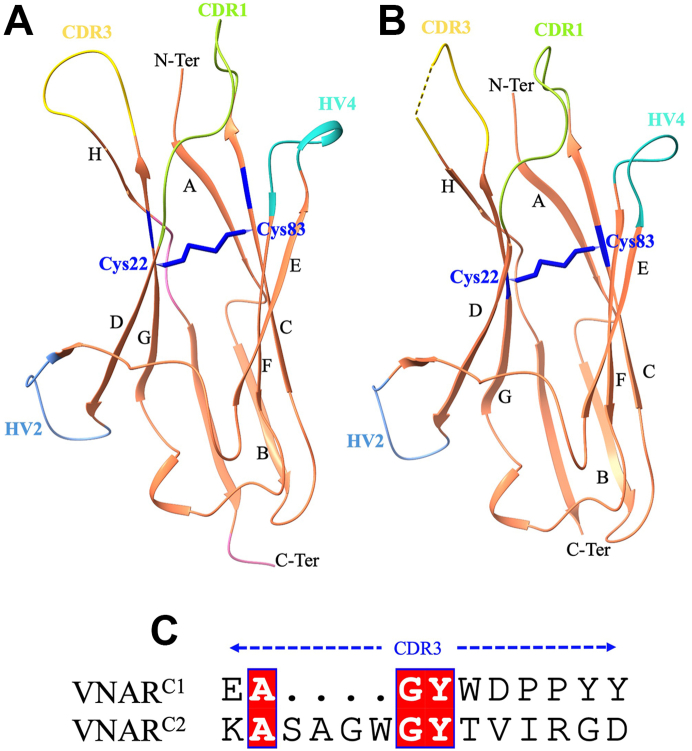
**Crystal structures of bamboo shark–derived vNAR nanobodies.** Structures of vNAR^C1^ (*A*) and vNAR^C2^ (*B*) with hypervariable regions highlighted. The hypervariable regions CDR1 (residues 25–33) and CDR3 (residues 87–96) are depicted in *green* and *yellow*, respectively. The HV2 (residues 44–51) and HV4 (residues 60–65) regions are shown as *light blue* and *cyan*, respectively. The disulfide bond is highlighted in *blue*. In vNAR^C2^, residues 93 to 97 of CDR3, indicated by a dotted gap, are disordered and are not included in the final model. *C*, sequence alignment of the CDR3 regions of vNAR^C1^ and vNAR^C2^ showing the sequence variability. CDR, complementarity-determining region; vNAR, variable new antigen receptor.

For structural studies, we selected two type IV vNAR nanobodies having different CDR3 length (one having 10 and the other having 14 amino acids) and were purified in a bacterial expression system with a purity of >95% (Fig. S9). The crystal structures of the vNAR^C1^ and vNAR^C2^ nanobodies were determined at a resolution of 2.0 Å and 1.8 Å, respectively, using a GFP14 (PDB ID: 8HGI) (11) as a search model for the molecular replacement method. Crystals of vNAR^C1^ (PDB ID: 9UP9) and vNAR^C2^ (PDB ID: 9UVH) belonged to the space groups *P* 4_1_2_1_2 and *P* 2_1_, respectively, each containing one molecule per asymmetric unit. Data collection and refinement statistics are summarized in Table 2. The crystal structures of our vNAR exhibit a compact immunoglobulin-like β-sandwich fold, comprising eight canonical β-strands arranged into two antiparallel β-sheets, hallmark features of shark-derived IgNARs (Fig. 6). The β-sandwich is stabilized by a canonical disulfide bond between residues Cys22 and Cys83 connecting strands G and C (Figs. 6 and S10). Consistent with the type IV vNAR subclass described by Zielonka *et al.* (2015) (12), these domains lack noncanonical disulfide bonds, with no evidence of framework-CDR or CDR1-CDR3 linkages, further supporting their classification as type IV. The CDR3 loop is markedly extended and structurally flexible, a defining feature of type IV vNARs, adopting a conformation well-suited for engaging cryptic or recessed epitopes. Electron density is well resolved for residues 2 to 107 in vNAR^C1^ and vNAR^C2^ structures, except for partial disorder in the CDR3 region (93–97) of vNAR^C2^, suggesting greater flexibility in the CDR3 of vNAR^C2^.

**Table 2:** Data processing and refinement statistics

Parameter	VNAR^C1^ (PDB ID: 9UP9)	VNAR^C2^ (PDB ID: 9UVH)
Resolution range	28.21 - 2.0 (2.072 - 2.0)^a^	19.95 - 1.8 (1.864 - 1.8)^a^
Space group	*P* 4_1_ 2_1_ 2	*P* 1 2_1_ 1
Unit cell	64.70 64.70 57.59 90 90 90	33.57 34.48 36.04 90,109.952 90
Multiplicity	17.7 (12.2)	9.6 (7.7)
Completeness (%)	99.62 (99.53)	99.86 (100.00)
I/σ(I)	15.95 (1.66)	33.92 (8.73)
Wilson B-factor	28.43	12.99
R_merge_^b^	0.17 (1.2)	0.07 (0.21)
R_pim_	0.04 (0.41)	0.02 (0.08)
CC1/2	0.98 (0.26)	0.99 (0.97)
Reflections used in refinement	8672 (841)	7310 (720)
Reflections used for R-free	433 (38)	355 (35)
R_work_^c^	0.21 (0.30)	0.16 (0.15)
R_free_^c^	0. (0.27)	0.20 (0.18)
Number of nonhydrogen atoms	880	851
Macromolecules	830	784
Ligands	4	
Solvent	46	67
Protein residues	106	103
RMS (bonds) (Å)^d^	0.011	0.015
RMS (angles) (˚)^d^	1.81	2.21
Favored (%)	99.04	98.99
Allowed (%)	0.96	1.01
Outliers (%)	0.00	0.00
Rotamer outliers (%)	0.00	1.18
Clashscore	0.61	0.65
Average B-factor (Å^2^)	35.16	16.29
Macromolecules (Å^2^)	35.11	15.73
Solvent (Å^2^)	35.91	22.84

a Values in parentheses are for the highest resolution shell.

b *R*_merge_ = Σ| *I* - ⟨*I*⟩|/Σ*I*.

c *R* = Σ|*F*_obs_| - |*F*_calc_|/Σ|*F*_obs_|. The *R*_free_ is the *R* calculated on the 5% reflections excluded for refinement.

d RMS is root mean square.

### Comparative analysis of the shark type IV vNAR crystal structures with its homologs

**Figure 7:**
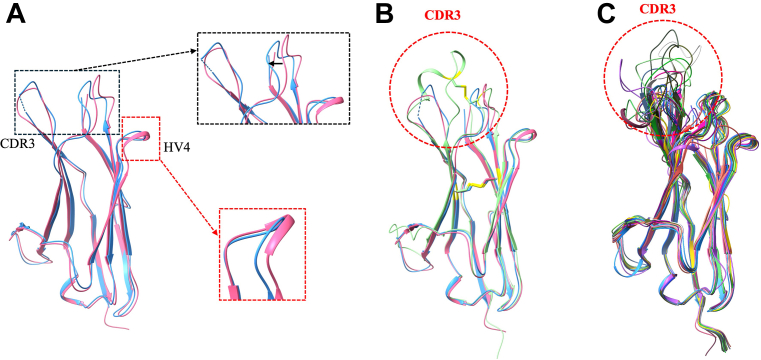
**Structural comparison of bamboo shark vNAR domains with homologous nanobodies.***A*, superimposition of vNAR^C1^ and vNAR^C2^ structures, highlighting conformational differences in the CDR3 and HV4 regions (indicated by *black* and *red* boxes, respectively). *B*, structural alignment of vNAR^C1^ and vNAR^C2^ with the homologous nanobody aGFP14 (PDB ID: 8HGI) reveals a notable change in the CDR3 region. *C*, structural alignment of 22 AlphaFold-predicted vNAR models with the experimentally determined crystal structure shows conserved framework regions (RMSD < 0.8 Å) and significant variability in the CDR3 region. CDR, complementarity-determining region; vNAR, variable new antigen receptor.

Structural comparison of vNAR^C1^ and vNAR^C2^ (RMSD 0.7 Å) revealed that the most pronounced conformational differences were localized within the CDR3 region (residues 87–96) (Fig. 7*A*). In vNAR^C2^ CDR3, residues Tyr 93 and Tyr 95 point upward, with residues 92 to 95 (PYYY) adopting a type II β-turn. Additionally, residues 62 to 65 form a single turn of a 3_10_ helix in vNAR^C1^ but not in vNAR^C2^. Comparison of vNAR^C1^ and vNAR^C2^ shark nanobodies with the closely related vNAR aGFP14 (PDB ID: 8HGI) revealed major differences in the CDR3 region (RMSD 0.7 Å). In aGFP14, CDR3 adopts a helical conformation and is extended by ∼6 to 10 residues, whereas in vNAR^C1^ and vNAR^C2^, it forms a shorter, flexible loop (Fig. 7*B*). Superimposition of all AlphaFold-predicted vNARs clone structures onto vNAR^C1^ and vNAR^C2^ structure revealed that some variants possess two disulfide bonds while others contain only one, highlighting the structural diversity present within the vNAR library (RMSD range 0.4–0.8) (Fig. 7*C*).

**Figure 8:**
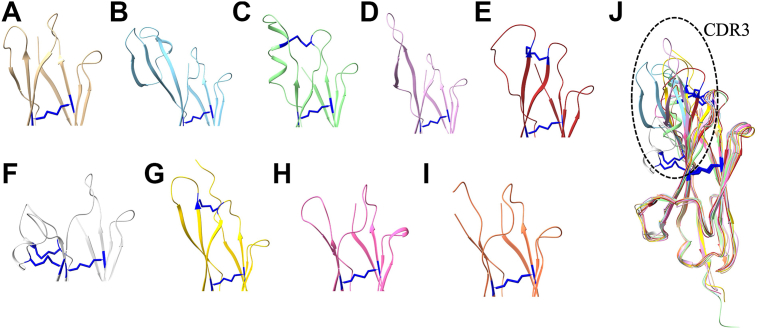
**Cartoon representations depicting structural variability in the CDR3 region of the vNAR domains.***A*, short loop (type IV, PDB ID: 4HGK); (*B*) large loop with one disulfide constraint (type IV, PDB ID: 3MOQ); (*C*) highly constrained loop tethered by two cysteine motifs (type II, PDB ID: 2I25); (*D*) extended CDR3 forming an α-helical motif (type IV, PDB ID: 2Z8V); (*E*) extended CDR3 forming a two-stranded β-sheet (type II, PDB ID: 2COQ); (*F*) extended CDR3 incorporating an amyloid-β p3 fragment (type I, PDB ID: 1SQ2); (*G*) AlphaFold-predicted model (type III, AAM76948 from Streltsov *et al.*); (*H*) short loop (type IV, PDB ID: 9UP9, this study); (*I*) short loop (type IV, PDB ID: 9UVH, this study); (*J*) superimposition of structures A–I, showing variability in the CDR3 region (highlighted by *black* circle). CDR, complementarity-determining region; vNAR, variable new antigen receptor.

Comparative structural analysis of vNAR^C1^ and vNAR^C2^ (type IV; PDB IDs: 9UP9 and 9UVH, this study) with previously reported vNAR types (I, II, III, and IV) highlights the distinct features of type IV domains (Fig. 8). Notably, the CDR3 regions in our structures display substantial sequence and conformational variability, a hallmark of antigen specificity. Unlike types I–III, which exhibit additional noncanonical disulfide bonds linking CDR loops, type IV vNARs lack these constraints, contributing to their extended loop flexibility. Our aligned structural representations further underscore the absence of interloop disulfide bridges in type IV domains, distinguishing them from the more constrained architectures of types I–III.

## Discussion

Nonimmune naïve nanobody libraries have broad applicability for isolating nanobodies against diverse antigens. However, constructing a highly diverse naïve nanobody library is a complex and labor-intensive task (1). Currently, shark-derived naïve vNAR libraries are not commercially available. Therefore, laboratories interested in this approach must either construct their libraries or work within the limitations of available alternatives. In the past, a few attempts have been made to construct immune shark libraries specific to selected antigens (13, 14, 15, 16, 17, 18, 19). Efforts have also been made to develop naïve vNAR libraries. However, most of the previously constructed naïve libraries exhibit limited diversity, typically containing 10^7^-10^8^ clones (9, 20). Notably, Kolmar's group developed a semisynthetic bamboo shark library with CDR3 randomization, though the resulting binders often required *in vitro* affinity maturation to achieve high affinities (5, 21).

The isolation of diverse high-affinity nanobodies and antibodies from a nonimmune naïve repertoire depends on the quality, size, and diversity of the constructed library (1). In comparison to immunized libraries, naïve libraries provide high diversity and can target a wide range of potential antigens (3). In the present study, we have addressed the limitations of current libraries by offering a larger and more diverse collection of all known four classes (I to IV) of vNAR nanobodies.

Notably, most of the currently available naïve phage display libraries analyze a limited number of clones based on the vector-to-insert ratio. The presence of desired inserts does not accurately represent the true functional diversity of the library. Additionally, libraries constructed with a lower vector-to-insert ratio were unable to isolate desired binders. A common challenge during the preparation of such libraries is the presence of bald clones, which carry inserts with either stop codons or frameshift mutations that hinder their functionality, collectively reducing the effective size of the library. Library diversity further diminishes as the wild-type pIII molecules on these bald phage selectively support their growth, causing them to outcompete others (22). To facilitate the selection of in-frame antibody repertoire sequences, an anti-HA tag antibody has been employed to proofread the panning process and select in-frame functional clones (23). Alternatively, in another study, a β-lactamase selection system was utilized to enrich the in-frame clones (24). However, in the current study, the occurrence of out-of-frame inserts was not observed as a single-round cloning strategy was employed during library construction. Furthermore, to reduce clones lacking insert sequences, we implemented stringent measures, such as reducing the number of self-ligated vector colonies by treating the cleaved vector DNA with Calf intestinal phosphatase and initiating the construction of sublibrary stocks after negligible colonies were detected post-transformation and plating, further minimizing even picomolar concentrations of uncut or self-ligated DNA (25). Various sublibraries were prepared, and the presence of functional vNAR clones in each sublibrary stock was validated through Western blot analysis, which confirmed that >85% of the clones exhibited detectable levels of vNAR expression. The remaining ∼15% of clones showing negative vNAR expression were hypothesized to have stop codons or frameshift mutations within their ORFs. To assess this, we selected random nonexpressing clones for Sanger sequencing. Sequencing data confirmed that the majority of these clones had proper insert sequences with functional ORFs. We further hypothesized that the observed lack of expression might stem from low levels of vNAR expression rather than genetic defects. To test this hypothesis, some of these expression-negative clones were cultured in larger volumes (200 ml) and subjected to protein expression, followed by purification using Ni-NTA affinity chromatography. As anticipated, most of these clones exhibited low levels of vNAR expression but were nonetheless expression-positive when cultured in large volumes. This observation confirmed that >90% of the clones in the library are capable of expressing functional vNARs. We performed next-generation sequencing (NGS) analysis of the central library to assess the diversity of our library, which revealed a diverse pool of naïve vNAR sequences. The diversity was predominantly observed in the CDR3 region, with >50% of clones exhibiting unique CDR3 sequences. These results align with previously published studies reporting that the primary vNAR repertoire in nurse sharks exhibits its highest diversity within the CDR3 region. Furthermore, somatic mutations have also been noted in other hypervariable regions, including CDR1, HV2, and HV4 (12, 26). In our study, ∼ 6% of the clones from the bamboo shark library exhibited sequence diversity in the CDR1 region. This finding is consistent with previous reports on the wobbegong shark, where limited diversity within the CDR1 region is documented (9). The vNARs gene sequences are categorized into four subtypes (type I–IV) based on the presence of cysteine residues. Previous studies conducted on bamboo sharks have reported that the majority of vNARs are either type I or type II (Table 3) (3, 27). However, it is noteworthy that these prior studies were based on immune display libraries, which could potentially influence the observed diversity patterns. In the current study, NGS data have shown the presence of all four types of vNAR (type I: 15.2%; type IIa: 23.9%; type IIb: 41.5%; type III: >1%; type IV: 19.3%) in the constructed naïve library, making it a valuable source to isolate high-affinity binders. In our study, the type II vNAR variant is the most predominant subtype, followed by type IV. The previous studies involving immune libraries from bamboo sharks reported minimal presence of type IV vNARs. Interestingly, approximately one-fifth of the clones express type IV vNARs in our study. The type IV vNARs are structurally distinct from other vNAR subtypes due to the lack of noncanonical disulfide bonds, providing greater structural flexibility to these vNARs and preventing physical constraints in their paratopic regions, which enable them to bind with cryptic epitopes that are typically inaccessible to other vNAR classes. Another novel feature of our library is the presence of atypical type IIb vNARs, containing Cys residues in either CDR1 or CDR3, which inhibit them from forming disulfide linkages (except for canonical) and thus they exhibit enhanced loop flexibility, structurally resembling type IV vNAR domains (9). Previous studies have documented that both type IV and atypical type IIb vNARs exhibit substantial structural homology to human immunoglobulin sequences, particularly within their framework regions. This homology facilitates the humanization of type IIb and type IV vNARs, potentially improving their biophysical properties for human clinical use (28, 29). Our library also showed diversity in the number of Cys residues within the CDR3 region (ranging from 0 to 5 Cys), which may contribute to a structurally diverse repertoire of unique clones exhibiting a wide variety of loop conformations capable of recognizing a broad spectrum of epitopes.

**Table 3:** Comparative analysis of available shark vNAR libraries

S. No.	Shark	Naïve/Immune	Diversity	vNAR type	Reference
1.	Bamboo shark	Immune	10^9^	Type I & III	(45)
2.	Banded wobbegong shark	Immune	1.16 × 10^6^	Type I 12% Type II 88%	(18)
3.	Bamboo sharks	Immune	10^7^	Type I 0%& Type II 79% Type III 0.03% Type IV 1.9%	(3)
4.	Bamboo sharks	Immune	1 × 10^9^	NA	(46)
5.	Bamboo sharks	Naïve/Immune	10^9^/10^8^	NA	(47)
6.	Spotted wobbegong sharks	Naïve	3 × 10^7^	NA	(34)
7.	Spiny dog fish sharks Smooth dog fish shark	Naive	1 × 10^7^	Type II 93% Type III 7%	(9)
8.	Nurse sharks	Naive	1.2 × 10^10^	Type I 23.5& Type II 56.7% Type III 0.005% Type IV 1.6%	(7)
9.	Bamboo shark	Naive	2 × 10^8^	NA	(5)
10.	Spotted Wobbegong sharks	Naive	4.0 × 10^8^	NA	(48)
11.	Bamboo Shark	Naïve	3 × 10^11^	Type I 15.2%& Type II 65.4% Type III 0.1% Type IV 19.3%	This Study

NA: vNAR type data not mentioned in the cited paper.

To gain structural insights into type IV vNARs, we determined the X-ray crystal structures of two vNAR clones with different CDR3 lengths: one with an average CDR3 (10 amino acids) and the other with an extended CDR3 region (14 amino acids). The crystal structures of these vNARs demonstrate conserved vNAR folds with flexible paratopic regions. Our study reported the isolation of high-affinity binders in the nanomolar range against a diverse set of antigens, reflecting a high hit rate of ∼100%. Along with that, based on the target antigens, about 30 to 100% of the binders were reported to be unique. The isolation of unique clones dropped during the fourth round of BP, while the maximum number of unique clones with substantial CDR3 variation was reported in the second and third rounds of BP. This pattern is consistent with most of the antigens (9). The BLI data reveal that vNAR nanobodies isolated against a diverse set of antigens showed varied affinity, ranging from micromolar to the picomolar range (30). Similar findings in the past have been validated on naïve human libraries where antibodies with nanomolar to picomolar affinities have been identified. Small libraries, with sizes ranging from 10^7^ – 10^8^, usually isolate binders of micromolar to low nanomolar range, and subnano to picomolar antibodies have been isolated from large-sized naïve human libraries with a diversity of 10^10^ to 10^11^ (31, 32, 33). However, no such reports are available on the characterization of vNAR nanobody naive libraries.

The efforts to construct naïve vNAR libraries, particularly in bamboo sharks, remain limited. Nuttall *et al.* reported the construction of a naïve library derived from wobbegong sharks, with a total diversity of ∼4.0 × 10^8^ (34). However, that library contained a pool of ∼7.0 × 10^6^ clones derived from natural cDNAs, while >3 × 10^8^ clones consisted of synthetic CDR3 sequences (20). Liu *et. al.* constructed naïve phage display libraries from spiny dogfish shark (*Squalus acanthias*) and smooth dogfish shark (*Mustelus canis*). These shark species are phylogenetically distant from nurse and wobbegong sharks, and the resulting libraries had an estimated library size of >10^7^ (9).

In conclusion, we developed a high-diversity functional naïve vNAR library, which yielded specific and potent binders against all the tested antigens (n = 9) during the panning process. To increase the technology readiness level, the library must now be panned against a greater number of diverse targets. An in-depth understanding of vNAR–antigen complexes will help design robust humanization strategies. At this point, this library is a good source to identify lead candidates for various biomedical applications.

## Experimental procedures

### Preparation of shark cDNA

The spleen of the white-spotted bamboo shark (*C. plagiosum*) was extracted and crushed in a cell stainer. The cells were washed thrice with 1X PBS to remove any residual fat cells and stored in Trizol (RNAiso Plus, Takara Cat. No. 9108/9109) till total RNA isolation was performed. RNA was isolated using Trizol following the manufacturer’s protocol. Prime Script 1^st^ strand cDNA Synthesis Kit (Takara, Cat. No 6110A) was used for cDNA preparation with 50 μg of total RNA.

### vNAR amplification

vNAR sequences were amplified from cDNA using two sets of reverse and forward primers (Table S1). Two different polymerases, that is, Phusion High-Fidelity DNA Polymerases (Thermo Fisher Scientific, Cat. No. F530L) and REDTaq ReadyMix PCR Reaction Mix (Sigma, Cat. No. R2523), were used for the amplification of vNAR sequences using gradient PCR with temperatures ranging from 55 °C to 62 °C.

### Digestion of vNAR and pSEX81

After PCR amplification, a 1.5% agarose gel was used to separate the amplified vNAR products. A distinct band corresponding to ∼400 base pairs (bp) was excised from the gel, and the PCR-amplified sequences were extracted from the excised gel fragment using the QIAquick Gel Extraction Kit (Qiagen, Cat. No. 28706). For 1 μg of purified PCR product, a 50 μl restriction digestion reaction was performed using *NcoI-HF* (NEB, Cat. No. R3193) and *NotI-HF* (NEB, Cat. No. R3189) restriction enzymes in rCutSmart buffer (NEB, Cat. No. B6004S) at 37 °C for 4 to 6 h. The pSEX81 vector was also digested with the same set of enzymes in the rCutSmart buffer. After 3 h of vector digestion, Quick Calf intestinal phosphatase (NEB, Cat. No. M0525 L) was added at a concentration of 1 μl/μg of digested vector for dephosphorylation, and the reaction was incubated for 1 h at 37 °C. Dephosphorylation was continued for an additional 2 h at 37 °C.

### Ligation of vNAR and pSEX81

The digested vector and PCR products were subjected to column purification using the QIAquick PCR Purification Kit (Qiagen, Cat. No. 28106). Phagemid vector, pSEX81 was used for the expression of functional recombinant vNAR - pIII fusion protein libraries on the surface of M13 filamentous phage. Two sets of ligation reactions were performed with an insert-to-vector ratio of 1:1 and 2:1 using the T4 DNA Ligase enzyme (Thermo Fisher Scientific, Cat. No. EL0012) in the appropriate buffer. The ligation reaction was maintained at room temperature and incubated overnight for a duration of 16 h at 16 °C. Subsequently, 5 μl of ligated products were transformed into chemically competent TG1 cells *via* heat shock, which involved a 30-min (min) cold treatment of the cells under ice, followed by incubation for 90 s in a water bath at 42 °C. Following heat shock, the TG1 cells were supplemented with 1 ml of super optimal broth recovery media and incubated for 1 h at 37 °C. After recovery, cells were harvested by centrifuging the culture at 8000 rpm for 5 min. Nine hundred microliters of the supernatant comprising SOC media was discarded, and the bacterial pellet was resuspended in the remaining 100 μl of SOC media and plated on 2xYT agar (Himedia, Cat. No. G035) plates supplemented with 20 mM glucose and 100 μg/ml of ampicillin. The plates were incubated overnight at 37 °C and were examined for bacterial colonies.

### Colony PCR and restriction digestion analysis

After overnight incubation, bacterial colonies were randomly picked from the 2xYT agar plates and subjected to colony PCR to confirm the successful insertion of the ligated products. The randomly selected colonies were dissolved in 10 μl nuclease-free water (Himedia, Cat. No. ML024) and heated at 95 °C for 15 min. The dissolved colony samples were centrifuged, and 5 μl of supernatant was used for PCR amplification of the vNAR sequences using REDTaq ReadyMix PCR Reaction Mix with appropriate forward and reverse primers. 1.5% agarose gel was used to analyze the PCR products to check the presence of a ∼400 bp insert. Validation of positive clones was also performed by restriction digestion analysis. Clones were randomly picked, grown in 2xYT broth (Himedia, Cat No. G034), harvested, and plasmids were isolated using QIAprep Spin Miniprep Kit (Qiagen, Cat. No. 27106) according to the manufacturer’s protocol. Subsequently, 2 μg of isolated plasmid was digested in a 50 μl restriction digestion reaction using the same set of restriction enzymes as described above. The digestion reaction was maintained at 37 °C for 2 h, followed by analysis of the digested fragments in a 1.5% agarose gel.

### Expression analysis

SDS-PAGE and Western blot were performed to check the vNAR expression profile of the positive clones. TG1 colonies from 2xYT plates were pooled into a single tube of 2xYT broth and allowed to grow overnight at 37 °C under shaking conditions. Cells were harvested and plasmid isolation was performed using QIAprep Spin Miniprep Kit. For expression studies, we have subcloned vNAR gene from phage ELISA positive clones to pHAL14 phagemid vector. This vector results in the higher expression of soluble vNAR nanobodies and scFvs as described in (35). A total of 5 μg of isolated plasmid was digested using *NcoI-HF* and *NotI-HF*, and the cleaved insert was ligated into pHAL14 expression vector containing a hexa-histidine tag. Five microliters of the ligated products were transformed in BL21 cells *via* the heat shock method as described above. Transformed cells were plated on ampicillin-supplemented 2xYT plates, and after overnight incubation, clones were randomly picked, inoculated in 2xYT broth, and protein expression was induced with 1 mM IPTG (Molychem, Cat No. 15130) when the cultures reached an OD_600_ of 0.4. After the addition of IPTG, cultures were incubated for 4h at 37 °C. This was followed by centrifugation of the cultures at 6000 rpm for 20 min, and bacterial pellets thus formed were resuspended in 8 M urea and incubated for 16 h. The solubilized pellets were centrifuged at 13,000 rpm for 10 min, and 30 μl of supernatant was mixed with 10 μl 4X SDS gel loading dye and heated at 100 °C for 15 min before being loaded into a 15% SDS gel. After the gel run was completed, it was stained with Coomassie brilliant blue (Himedia, Cat. No. MB153-25G), and protein content was checked by observing the gel under GelDoc. vNAR protein expression was further analyzed by Western blot, which involved the transfer of the separated proteins from the gel to a 0.2 μm polyvinylidene fluoride membrane (Biorad, Cat. No. 1620177). A constant current of 250 mA was provided for 2h during the wet electroblotting of the membrane. After the transfer was completed, the membrane was blocked with 5% BSA (Molychem, Cat. No.31280) for 1h at room temperature, followed by overnight incubation at 4 °C with mouse anti-His primary antibody (Invitrogen, Cat. No. MA1-21315) at a dilution of 1:2000. After primary incubation, the membrane was washed four times with 1x PBS supplemented with 0.1% Tween, followed by incubation with horseradish peroxidase (HRP)-labeled goat antimouse secondary antibody (Elabscience Cat. No. E-AB-1001,) at 1:10,000 dilution. The blots were developed using a chemiluminescent substrate (GBiosciences, Cat. No. 786-003).

### Library preparation

After functional validation on a small scale, the vNAR library was prepared. For this, multiple rounds of PCR amplification of vNAR were performed with Taq polymerase. Around 30 μg of amplified vNAR and vector was digested with *NcoI* and *NotI* restriction enzymes as described above. The digested vector and insert were subjected to column purification and quantified with the help of a nanodrop. Ligation was done in a 1:1 vector to insert ratio at 16 °C for 16 h as described above, and ligation products were column-purified and electro-transformed into TG1 electrocompetent cells. For electroporation, purified ligation products were added to 200 μl electrocompetent cells in a 2 mm Gene Pulser cuvette (Biorad, Cat. No. 165-2086). After the electric pulse, recovery media were instantly added to the cuvette, and cells were allowed to recover in a total volume of 5 ml for 40 to 50 min. After 1 h, cells were centrifuged, 4 ml supernatant was discarded, and pellets were resuspended in the remaining 1 ml recovery media. Ten microliters of the culture was serially diluted to obtain the library size; the rest of the culture was plated on a pizza plate and incubated overnight at 37 °C. Colonies were pooled in 5 ml of 2xYT media supplemented with 15% glycerol and stored at −80 °C for further use.

### NGS sample preparation

For NGS analysis, plasmids were isolated from naïve vNAR library. Phusion High-Fidelity DNA polymerase was used for the amplification of vNAR sequences using the primers mentioned in Table S1. The PCR protocol consisted of an initial denaturation at 98 °C for 30 s, followed by 10 cycles of 98 °C for 10 s, 55 °C for 30 s, and 72 °C for 45 s, with a final extension at 72 °C for 5 min. PCR products were run on a 1.5% agarose gel, and DNA bands at ∼400 bp were excised and purified using QIAquick Gel Extraction Kit according to the manufacturer’s protocol and then submitted for sequencing. To characterize the vNAR repertoire, sequencing was performed using the Illumina platform. FastQC was employed for quality control of the raw NGS data. For downstream protein-level analysis, we translated the nucleotide sequences into amino acid sequences using a transseq function from the Bioconductor package.

### BP and monoclonal phage ELISA

BP against viral and nonviral antigens was performed according to the protocol described in our previous publication (36, 37). Briefly, naïve vNAR central library was amplified in 2xYT broth supplemented with ampicillin and 1% glucose. At an OD_600_ of 0.4, 50 ml of this culture was infected with 2 × 10^11^ helper phages and incubated for 30 min at 37 °C. Subsequently, the culture was centrifuged, and the pellet was resuspended in 100 ml of 2xYT media containing 0.1% glucose, ampicillin, and kanamycin and incubated overnight at 30 °C. Following incubation, the culture was centrifuged at 6000 rpm for 20 min, phage particles were precipitated using PEG/NaCl, and the phage titer was calculated. The rescued phages were used for the first round of panning. Four rounds of panning were performed with various antigens, starting at a concentration of 5 μg/ml. With each successive round of panning, the antigen concentration was reduced, and the number of washings was increased to obtain high-quality binders.

Screening for potential binders was performed after the second, third, and fourth rounds of panning. Clones were randomly picked and allowed to grow in 2xYT media with ampicillin and 1% glucose. Culture was subjected to Helper phage infection at an OD_600_ of 0.4. Subsequently, the culture was maintained at 37 °C for 30 min and then centrifuged. The supernatant was discarded, and the pellet was dissolved in 5 ml of 2xYT media supplemented with ampicillin, kanamycin, and 0.1% glucose. The culture was incubated overnight at 30 °C. For monoclonal phage, ELISA antigen was diluted to 1 μg/ml in bicarbonate coating buffer, added to 96-well ELISA plates, and incubated overnight for efficient coating. Plates were washed with PBS, blocked with 5% nonfat milk for 1.5 h, and then washed thrice with PBS. Hundred microliters of phage diluted in 5% nonfat milk (1:1) was added to each well and incubated for 1 h. Plates were then washed four times with 0.1% PBST, followed by the addition of HRP-conjugated secondary anti-M13 antibody (Progen, Cat No. 61097-HRP) for 1 h. Finally, plates were washed with 0.1% PBST, and 100 μl of TMB substrate (Life Technologies Cat No. 02051241) was added to each well. Plates were incubated in the dark until color development was observed. Reaction was stopped with 2N H_2_SO_4_, and absorbance was measured at 450 nm.

### BLI for affinity determination

BLI experiments were performed to calculate the binding affinity of nanobody–antigen complexes. Most of the antigens used in BP contain Histidine-tag, and nanobodies also have Histidine-tag. To access binding affinity, we converted nanobody into nanobody-Fc format. Briefly, Human Fc sensors (ForteBio Inc.) were used to capture the nanobody Fc at 10 μg mL^-1^ as described previously in published papers from our lab (32, 38, 39). The BLI-binding experiment was performed in buffer [PBS, pH 7.4, 0.01% (w/v) BSA and 0.002% (v/v) Tween-20]. The antigen concentration was started from 500 nM and subsequent two-time dilution was done. Associations for most of the antigens were kept 120 s and dissociations were kept 600 s. Data was analyzed using the software ForteBio Data Analysis. All the experiments were at least repeated two times.

### Purification and crystallization

The vNAR clone plasmids were transformed into BL21 cells using the heat shock method, and the transformed cells were plated on 2xYT plates supplemented with ampicillin and incubated overnight. A single colony from transformed plates was randomly picked and inoculated in 2xYT broth. Protein expression was induced with IPTG as described above. Subsequently, the induced culture was centrifuged at 6000 rpm for 20 min, and the resulting pellet was resuspended in 1x PBS with a pH of ±1 unit of the pI of the vNAR protein. The cell suspension was subjected to two rounds of sonication, with each round lasting 30 min, and the lysate was clarified by centrifugation. The supernatant was collected and passed through an Ni-NTA column. Two wash buffers containing 10 mM and 30 mM imidazole were used to wash out nonspecific proteins. Target His-tagged protein was eluted using a high imidazole concentration of 300 mM, and the eluted protein fraction was then analyzed using SDS-PAGE. To further purify the protein sample and ensure monodispersity, size-exclusion chromatography was performed. The protein fraction eluted after Ni-NTA chromatography was passed through a Superdex column (Superdex 200 pg), leading to the removal of protein aggregates and other impurities. Elution profiles were monitored at 280 nm, and fractions were collected and analyzed by SDS-PAGE for purity.

Size-exclusion chromatography yielded high-purity, monomeric vNAR nanobodies, which were subsequently concentrated to ∼10 mg/ml using Amicon Ultra centrifugal filters with a 10 kDa molecular weight cutoff. Crystallization was carried out at 293 K using the sitting-drop vapor diffusion method in 96-well plates with commercially available crystallization screens (Hampton Research and Molecular Dimensions), with equal volumes (1 μl each) of protein and reservoir solutions (40, 41). vNAR^C1^ crystals were obtained from a condition containing 20% (w/v) PEG 8000 and 0.1 M Ches (pH 9.5) (A7, JCSG + screen; Molecular Dimensions), while vNAR^C2^ crystallized in 20% (w/v) PEG 10,000 and 0.1 M Hepes (pH 7.5) (H2, Crystal Screen; Hampton Research). Crystals were cryoprotected using the respective reservoir solution supplemented with 20% ethylene glycol and flash-cooled in liquid nitrogen at 100 K for data collection.

### Diffraction data collection, model building, and refinement

The X-ray diffraction data were collected at 100 K using a HPC (Hybrid Photon Counting) detector and at 1.541 Å wavelength at the Home Source facility of the Macromolecular Crystallography Unit, IIC, IIT Roorkee, India. Data collection and processing statistics are summarized in Table 2. Data quality was assessed using Aimless from the CCP4i2 suite (42). The vNAR^C1^ and vNAR^C2^ crystal structures were solved by molecular replacement using aGFP14 (PDB ID: 8HGI) as a search model (11). The model building and refinement were performed using several cycles of REFMAC to final R_cryst_ and R_free_ values to acceptable levels (43). Water molecules were added during the final stages of refinement. Structural figures were generated using ChimeraX (44) and pymol (https://www.scienceopen.com/document?vid=4362f9a2-0b29-433f-aa65-51db01f4962f).

## Data availability

Data will be made available on request.

## Supporting information

This article contains supporting information.

## Conflicts of interest

IIT Roorkee has filed a provisional patent application in India (Indian Patent Application No. 202511021258; Filed on: March 10, 2025). All other authors declare that they have no conflicts of interest with the contents of this article.
