# Supplementary material for: A high diversity naïve variable new antigen receptor, vNAR, phage library for rapid nanobody discovery across diverse antigens
Source: J Biol Chem. 2025 Dec 22;302(2):111083. doi: 10.1016/j.jbc.2025.111083 (PMC12865624; doi:10.1016/j.jbc.2025.111083)
Supplement: Supporting file [file mmc1.docx]

**Supporting figure 1**


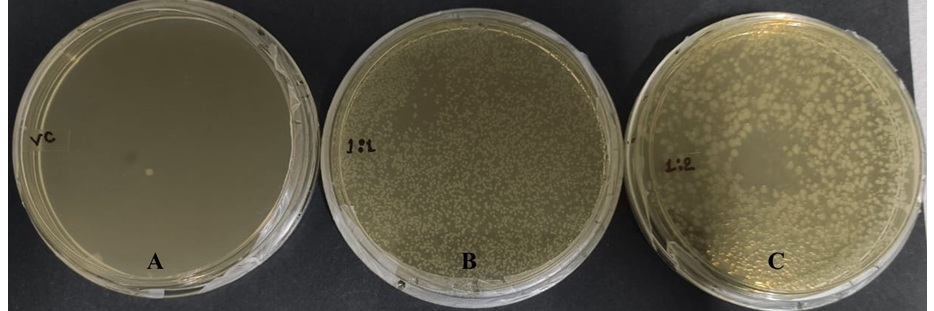


**Supporting figure 1:** *Optimization of Ligation ratio. Ligation reaction was performed in 1:1 and 1:2 vector to insert ratio. Plate B and C clearly indicate that the number of transformants were more in 1:1 ratio as comparison to 1:2 ratio. Plate A represents the vector control ligation, in which cut vector only was ligated and transformed.*

**Supporting figure 2**

**
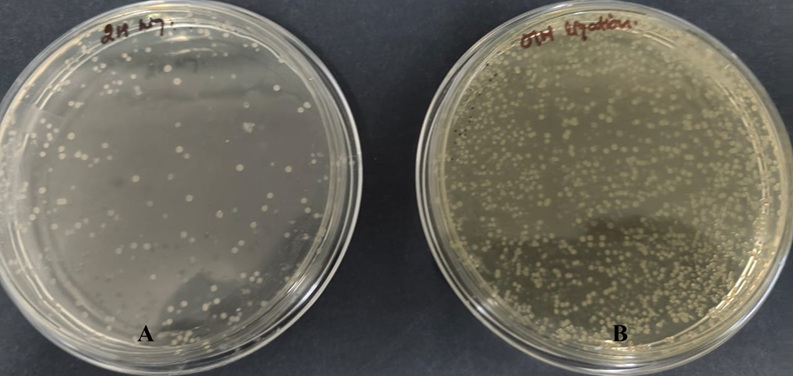
**

**Supporting figure 2:** *Optimization of Ligation time. Ligation reaction was performed at two different time period for maximum ligation efficiency. Plate A and B represent the number of transformants after one hour and overnight ligation reaction incubation time respectively. The data represents higher number of transformants were observed in overnight ligation reaction set up.*

**Supporting figure 3**

**
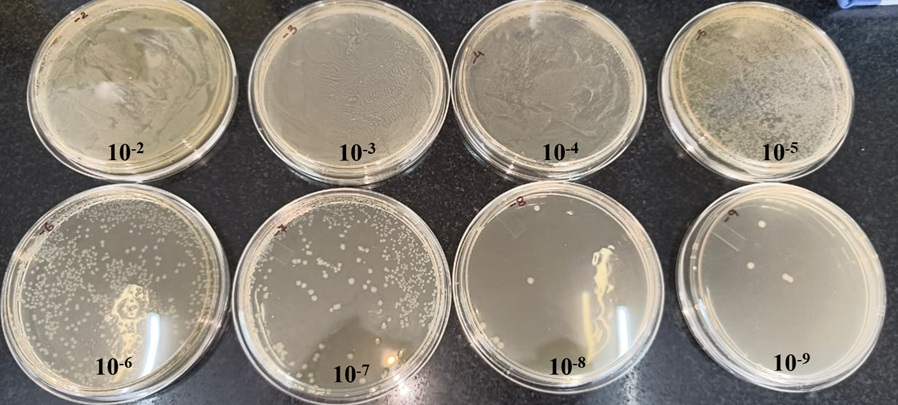
**

**Supporting figure 3:** *Size determination of Naïve shark library was determined by serial dilution method. After electro transformation, transformed cells are allowed to be recovered for 40 to 50 min, cells were centrifuged and dissolved in total 3 ml of media and then 10 µl of it was used for library size determination via serial dilution ranging from 10^-1^ to 10^-9^. Around 10 and 4 colonies were observed in 10^-8^ and 10^-9^ plate respectively. We have taken 10^-8^ dilution plate for size determination by the following formula*

*No. of transformants= No. of colony X 1000/ volume taken for titration x dil. Factor*

*=10 X 1000/10 X 10^-8 =^ 1 X 10^11^ / ml*

*Since, 3 ml of culture was there hence, Library size 3 X 1 X 10^11^ / ml = 3 X 10^11^*

**Supporting figure 4**


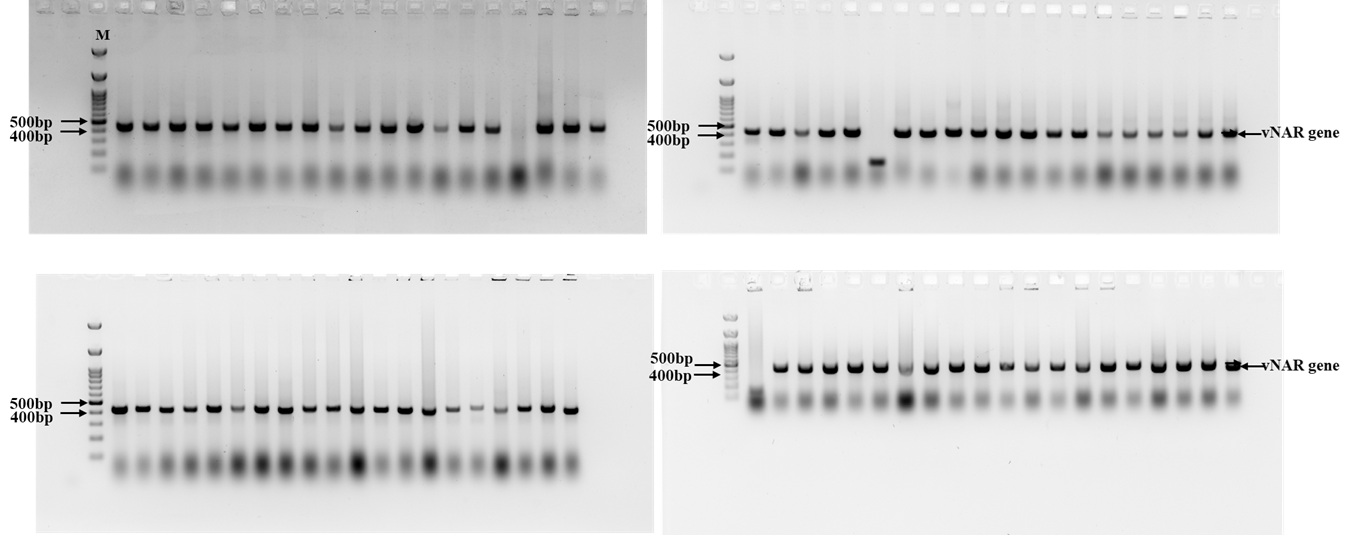


**Supporting figure 4: Representative images of colony PCR from randomly selected clones from different sub libraries.** *Analysis of randomly selected vNAR clones by colony PCR. Lane M represents the 100bp marker and product size around ~400bp in successive lane confirms the presence of vNAR 97% positive clones* (76/79).

**Supporting figure 5**

**
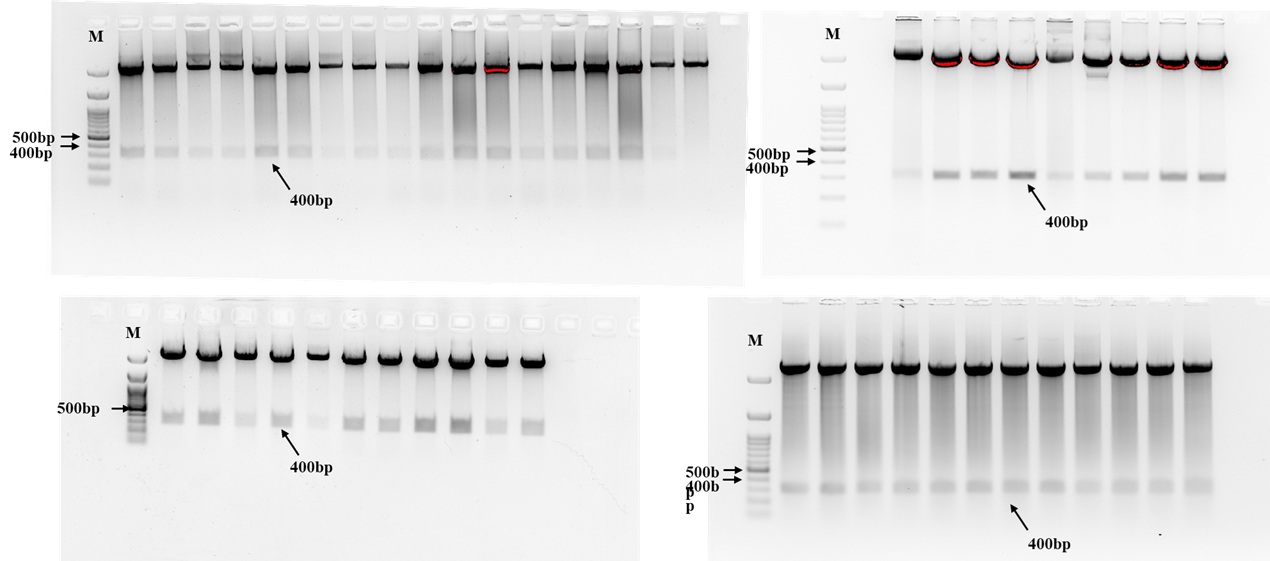
**

**Supporting figure 5:** **Representative images of restriction digestion analysis from randomly selected clones from different sub libraries.** *Restriction digestion Analysis of randomly selected vNAR clone by Agarose gel electrophoresis. Lane M represents the 100bp marker and inserts size around 400bp in successive lane confirms the positive clones (49/50)*

**Supporting figure 6**


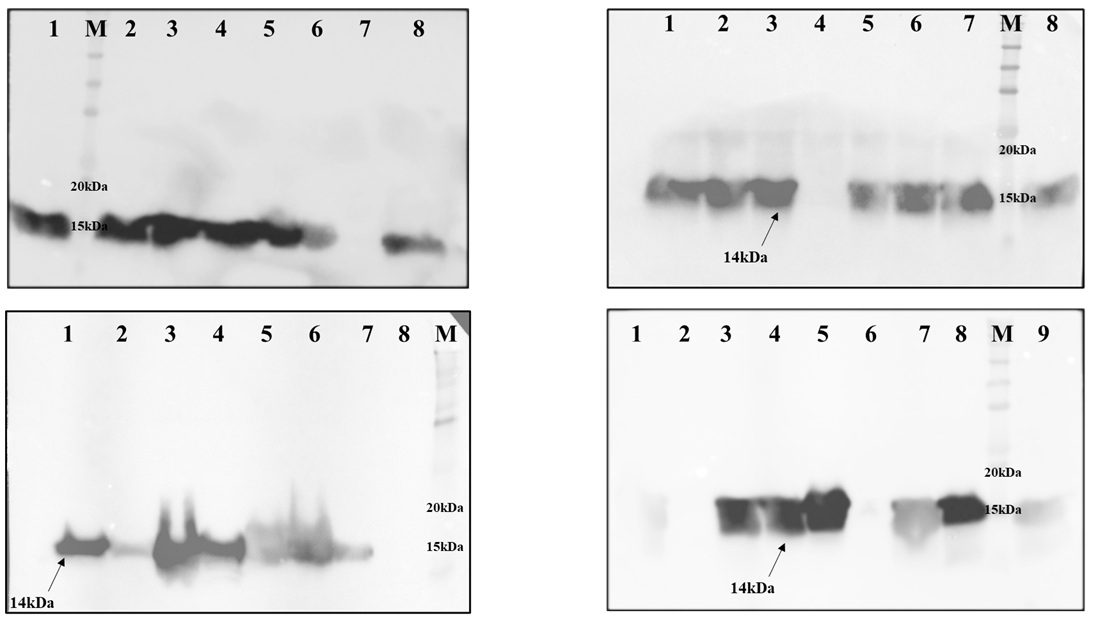


**Supporting figure 6:** **Representative images of expression profile analysis from randomly selected clones from different sub libraries.** *Expression analysis vNAR clones. Clones. Clones were randomly picked western blot was performed with Anti His primary antibody. A band around ~14kDa confirms the positive clones. 27 out of 32 clones were expression positive (~85%).*

**Supporting Figure 7**

*
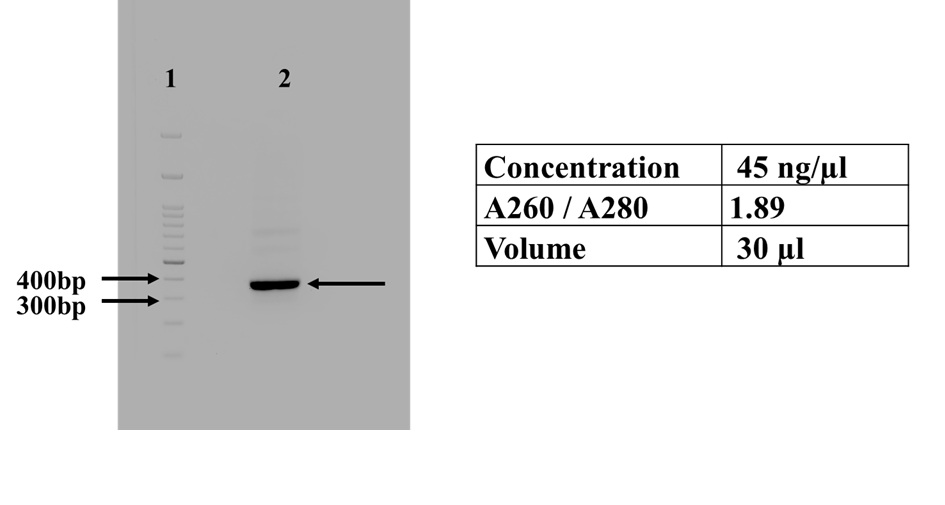
***Supporting Figure 7.** ***Sample preparation for NGS:*** *Diversity of the phage library was validated by sequencing using the Illumina platform. Plasmid DNA isolated from the central phage library stock underwent 10 PCR amplification cycles using the gene-specific primers. PCR product were separated on agarose gel (2 %), purified, and submitted for NGS analysis. Lane 1: 100 bp DNA ladder; lane 2: amplified PCR product. DNA purity and quality were confirmed by measuring the 260/280 absorbance ratio.*

**Supporting Figure 8.**

**
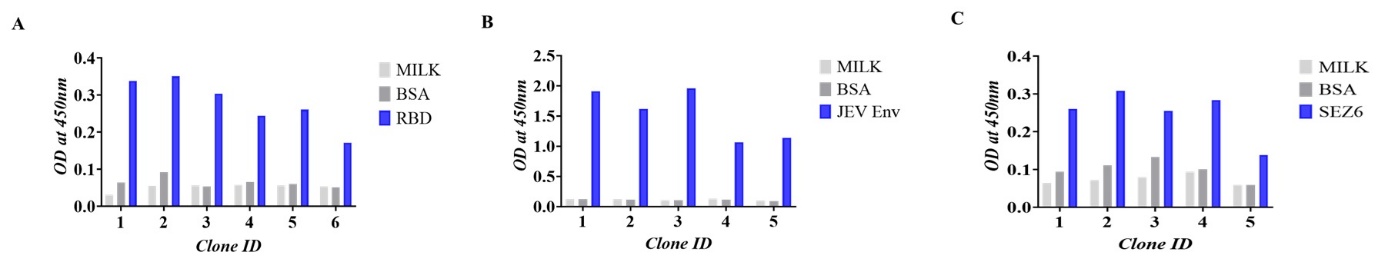
**

**Supporting Figure 8. Soluble ELISA of vNAR antigen binding clone to assess their functionality:** *The functional activity of phage ELISA antigen-positive clones was assessed by expressing vNAR in soluble form in an indirect ELISA. Represent binding of soluble vNAR clones to their respective antigens (A). RBD, (B). JEV Envelop (C). Lung cancer antigen. BSA and milk-coated wells were used as experimental negative controls. The ELISA experiment was repeated at least two times in duplicate.*

**Supporting Figure 9**


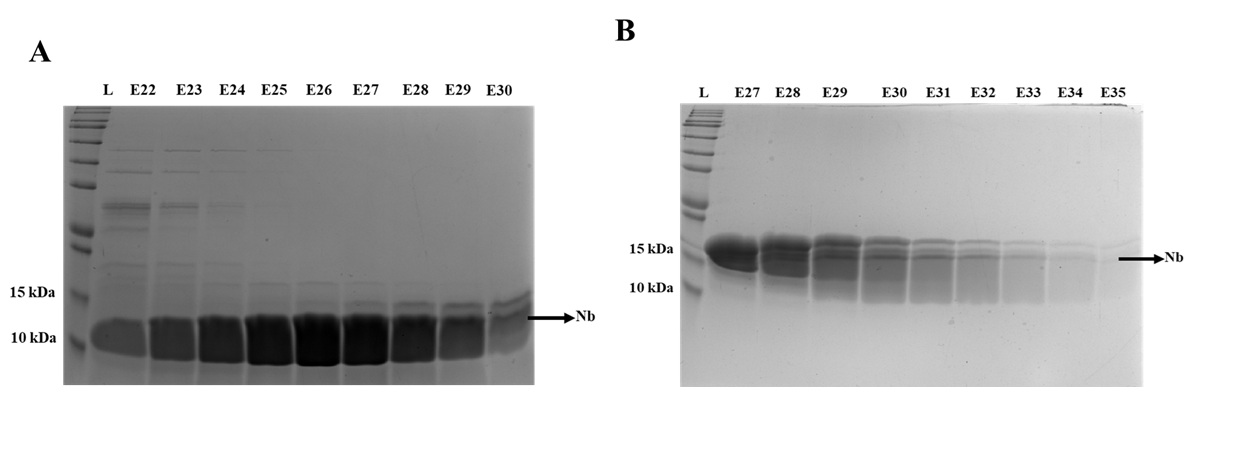


**Supporting Figure 9. Expression and purification of vNAR clones:** *vNAR nanobodies (A),vNAR^C1^ (B), vNAR^C2^  were purified from the soluble fraction of BL21(DE3). The vNAR clones were purified using Ni-NTA affinity chromatography. Lanes 1 to 9 represent different elute fractions. Lane M represents the protein ladder used for size determination.*

**Supporting Figure 10**


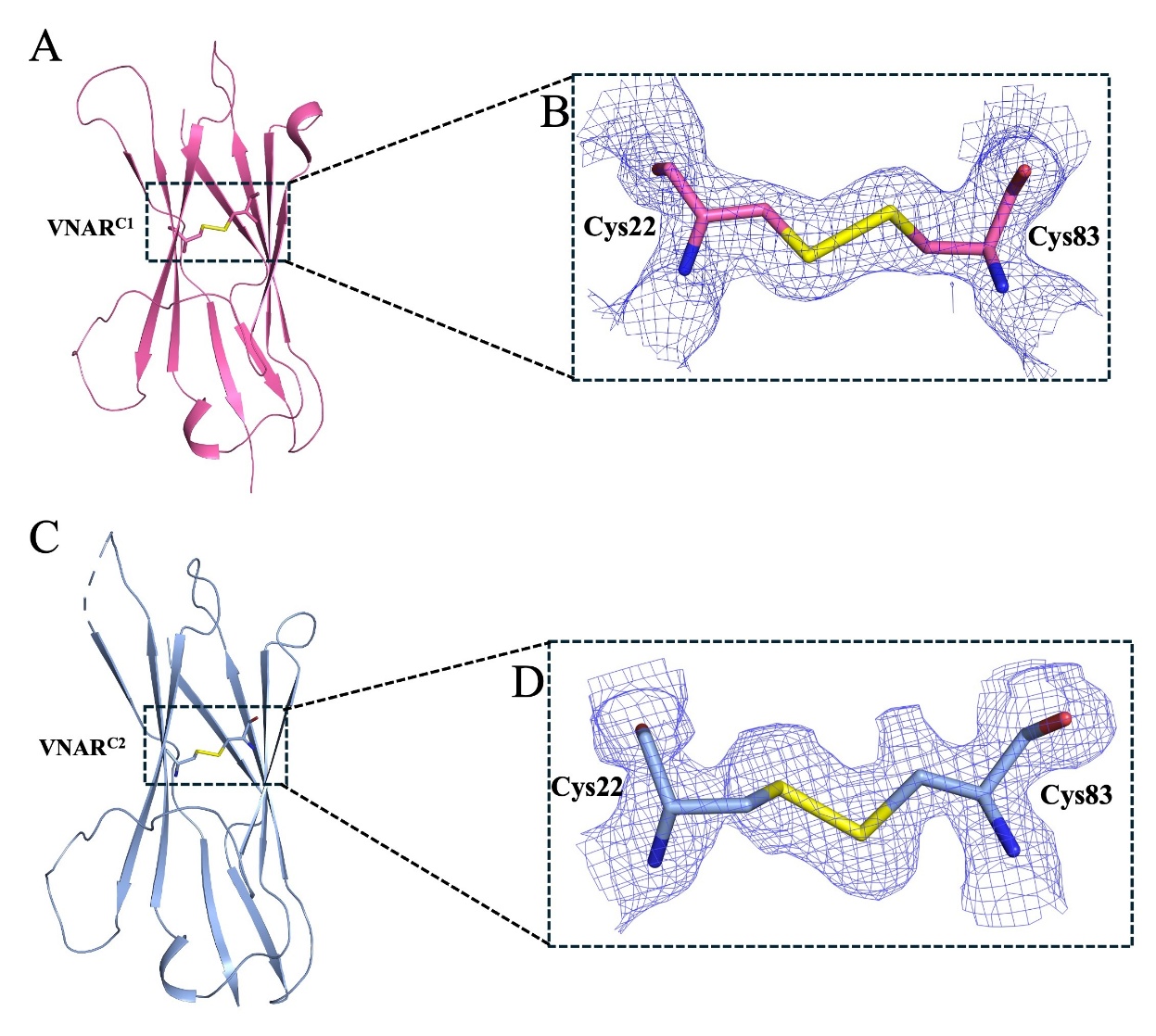


**Supporting Figure 10.** ***(A)*** *Crystal structure of vNAR^C1^ showing the canonical disulfide bond between residue Cys 22 and Cys 83.* ***(B)*** *Corresponding Fo–Fc omit map for the disulfide bond in vNAR^C1^, contoured at 1σ at 2.0 Å resolution.* ***(C)*** *Crystal structure of vNAR^C2^ highlighting the canonical disulfide bond between residue Cys 22 and Cys 83.* ***(D)*** *2Fo–Fc omit map for the disulfide bond in vNAR^C2^, contoured at 1σ at 1.8 Å resolution.*

**Supporting Table 1:** List of the primers used in the study. The restriction sites are highlighted.

| vNAR-1F | GTCCTCGCA***CCATGG***CCAACGGGTTGAACAAACACC |
| --- | --- |
| vNAR-2F | GTCCTCGCA***CCATGG***CGCATGGGTTGAGCAAACACCG |
| vNAR-1R | ACCGCCTCC***GCGGCCGC***TTTCACAGTCAGAATGGTGC |
| vNAR-2R | ACCGCCTCC***GCGGCCGC***TTTCACTGTTAGAAAAGTGCC |
